# Male age is associated with extra-pair paternity, but not with extra-pair mating behaviour

**DOI:** 10.1038/s41598-018-26649-1

**Published:** 2018-05-30

**Authors:** Antje Girndt, Charlotte Wen Ting Chng, Terry Burke, Julia Schroeder

**Affiliations:** 1 Evolutionary Biology, Max Planck Institute for Ornithology, Seewiesen, Germany; 2 Department of Life Sciences, Imperial College London, Silwood Park Campus, Ascot, United Kingdom; 3 International Max-Planck Research School (IMPRS) for Organismal Biology, University of Konstanz, Konstanz, Germany; 4 Department of Animal and Plant Sciences, University of Sheffield, Sheffield, United Kingdom

## Abstract

Extra-pair paternity is the result of copulation between a female and a male other than her social partner. In socially monogamous birds, old males are most likely to sire extra-pair offspring. The male manipulation and female choice hypotheses predict that age-specific male mating behaviour could explain this old-over-young male advantage. These hypotheses have been difficult to test because copulations and the individuals involved are hard to observe. Here, we studied the mating behaviour and pairing contexts of captive house sparrows, *Passer domesticus*. Our set-up mimicked the complex social environment experienced by wild house sparrows. We found that middle-aged males, which would be considered old in natural populations, gained most extra-pair paternity. However, both, female solicitation behaviour and subsequent extra-pair matings were not associated with male age. Further, copulations were more likely when solicited by females than when initiated by males (i.e. unsolicited copulations). Male initiated within-pair copulations were more common than male initiated extra-pair copulations. To conclude, our results did not support either hypothesis regarding age-specific male mating behaviour. Instead, female choice, independent of male age, governed copulation success, especially in an extra-pair context. Post-copulatory mechanisms might determine why older males sire more extra-pair offspring.

## Introduction

A robust finding in studies of avian extra-pair paternity is that older males sire more extra-pair offspring than younger males (see meta-analyses in^1,2^). What gives older males the competitive edge over younger males is unclear, but the finding has been considered to support the good genes’ hypothesis because older males have proven their viability and are considered to be of high genetic quality (reviewed by^3,4^). Females might seek copulations from older males to obtain genetic benefits for their offspring, but see^5^ for a critique. However, there is opposing, albeit inconclusive, empirical evidence for the idea that females gain genetic benefits through extra-pair mating^3,6^.

Extra-pair behaviour involves at least three individuals: the social male, the social female and one extra-pair male. The proximate mechanisms responsible for the positive association of male age with extra-pair paternity are unclear. It has been suggested that older males might outcompete younger males for extra-pair mating opportunities^7,8^ or that females may simply prefer older males as extra-pair partners^9,10^. Alternatively, older males might outcompete younger males post-copulatory through better sperm competition^11^. Here, we test whether older males achieve more extra-pair copulations and paternity, and whether female solicitation is associated with extra-pair mating.

Older males might be more experienced than younger males and better at convincing or forcing females to mate with them. Hence, older males are predicted to obtain more extra-pair copulations than younger males. This was coined the male manipulation hypothesis^12^. Through coercive mating, older males are also predicted to achieve more within-pair copulations^13^. Measuring the frequency of extra-pair copulations in wild populations, especially in non-colonial breeding birds, is difficult because extra-pair copulations can be secretive^14^. Several studies have analysed the copulation frequency or display rates of males in relation to their age in birds, e.g.^11,15^ and primates^16,17^. However, we are aware of only one study on the relationship between extra-pair copulations and male age; this showed that extra-pair mating attempts did not correlate with the estimated age of male razorbills, *Alca torda*, (*N* = 15 males)^18^.

The pattern of older males gaining more extra-pair paternity could also be caused by the mating behaviour of the female. The female choice hypothesis is supported by theoretical analysis^19^ but less so by empirical evidence: while a meta-analysis found some support for female birds preferring to copulate with older males^20^, a follow-up review reported mixed results^21^. The female-choice hypothesis is commonly tested by using extra-pair offspring as a proxy, e.g.^2^, instead of measuring female choice directly, but see^22^ for a behavioural approach in the wild. Using the number of extra-pair offspring as a proxy is a limitation because it reflects only the extra-pair copulations that led to fertilisation, but not how female choice for older extra-pair males is expressed behaviourally. For instance, females could either resist extra-pair mating attempts by older males until the costs of resistance are too great, and hence adopt a convenience polyandry strategy *sensu*^13^, or they might actively solicit extra-pair copulations from older males.

We used a captive population of house sparrows, *Passer domesticus*, of known ages to distinguish between those different strategies. We studied the copulation behaviour of both males and females in a semi-natural set-up. House sparrows are socially monogamous but sexually promiscuous. This means that a male and a female stay together for one, or more often multiple, breeding attempt(s)^23^, but copulations with an individual other than the social mate are evident from paternity analyses^2^. Further, male age is the most robust predictor of extra-pair paternity in house sparrows^2^.

In our set-up, males and females were kept in communal groups to mimic the gregarious colony structures found in wild house sparrow populations^23^. This laboratory environment has the advantage that females can choose among multiple males for within- and extra-pair mating and copulation behaviour can be measured. We first studied the association between extra-pair paternity and male age. We then tested the following predictions from the male manipulation, and female choice hypotheses, and also whether realised extra-pair paternity is a good proxy for copulation behaviour:

(1) We predicted that extra-pair paternity is positively associated with male age. (2) If older males are better at creating extra-pair mating opportunities, then we further predict that older males have more extra-pair copulations. (3) We predict that females solicit more often to older than younger males for both within-and extra-pair copulations and that female solicitation increases the probability of both within- and extra-pair copulations. Finally, (4) we tested the prediction that the number of extra-pair offspring correlates with extra-pair copulation behaviour. We also tested whether realised extra-pair paternity is a good proxy for copulation behaviour.

## Results

### Male age and its association with extra-pair paternity

Across the 400 embryos produced by 75 social pairs in four aviaries, 40 were extra-pair (i.e. 10% of all offspring). This value is slightly lower than a recent report on a wild house sparrow population, where 17.5% of all young were extra-pair^24^. Across broods (*N* = 119), 25 broods contained at least one extra-pair offspring (i.e. 21% of all broods) and there was no temporal association between laying date and extra-pair paternity (*N* = 40 extra-pair offspring, Spearman rank correlation, *rho* = 0.04, *P* = 0.79).

**Figure 1:**
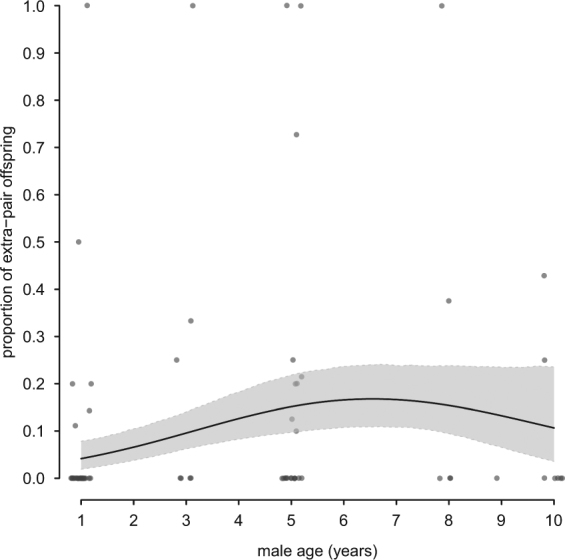
Proportion of extra-pair offspring in relation to the age of male house sparrows (*N * =75 males). Middle-aged males sired most extra-pair offspring. We show the average population regression line from the GLM (black line) with CrI (grey area). Open circles represent individual data offset at the x-axis to aid visualization.

We found that the frequency of extra-pair paternity and male age showed a statistically significant and non-linear relationship in our population: middle-aged males (i.e. 5 years old) sired the highest proportion of extra-pair offspring (Table 1, Fig. 1), e.g. 15% of middle-aged males’ offspring were extra-pair. Note that the result of older males achieving most extra-pair paternity remained qualitatively similar when running a zero-inflated Poisson but the quadratic age term was no longer significant (full model output in the Supplementary Information Table S1).

**Table 1:** The proportion of extra-pair paternity showed a statistically significant quadratic relationship with male age (*N* = 75 males).

	estimate (lower CrI to upper CrI)
**Fixed effects**	
(intercept)	−1.50 (−2.11 to −0.88)
male age	**0.81** (**0.31 to 1.32)**
male age^2^	**−0.61** (**−1.06 to −0.16)**
aviary B	0.18 (−0.70 to 1.06)
aviary C	−0.38 (−1.39 to 0.61)
aviary D	**−1.15** (**−2.20 to −0.10)**

Results are from a generalised linear model, GLM, assuming a binomial error distribution (logit-link function). Male age was centred and scaled. Extra-pair to within-pair offspring was fitted as a column-bound matrix proportional response variable. We show the model’s posterior means and 95% Credible Intervals (CrI). CrIs interpreted as statistically significant are in bold.

### Male manipulation hypothesis

**Figure 2:**
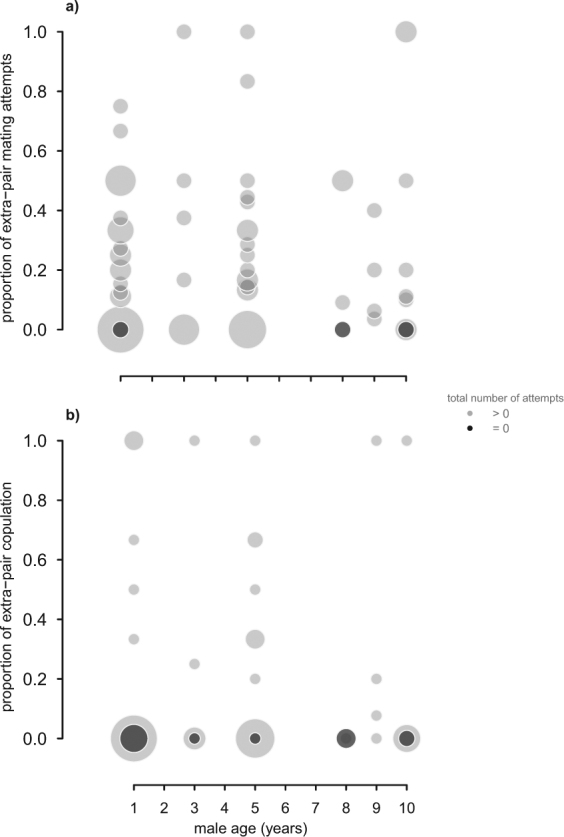
Extra-pair mating behaviour in relation to age in male house sparrows. Neither the proportion of extra-pair mating attempts (**a**) (*N* = 73 males) nor the proportion of extra-pair copulations (**b**) (*N* = 74 males) was explained by the age of males. Circles represent individual data and are scaled according to the number of males of a certain age that were (light grey) or were not observed (dark grey) as sexually active.

We observed a total of 463 mating attempts, ranging from 0 to 28 per male, and confirmed the occurrence of copulation, solicitation and the identities of the male and female in 425 of these 463 mating attempts (i.e. 91.8% observations). 107 male mating attempts were directed towards an extra-pair female. Male age did not predict the proportion of extra-pair mating attempts (estimated effect size 0.07 (CrI: −0.19 to 0.33, *N* = 73 males, Fig. 2a, Supplementary Information Table S2a). Further, we observed a total of 170 copulations, ranging from 0 to 13 per male. Of these, 27 copulations were with an extra-pair female. Similar to mating attempts, male age did not affect the proportion of extra-pair copulations (estimated effect size 0.03 (CrI: −0.51 to 0.57, *N* = 74 males, Fig. 2b, Supplementary Information Table S2b). Additionally, male age was not associated with the total number of mating attempts or copulations (Supplementary Information Table S3). Notably, 29 of 174 individuals (nine males and 20 females) were never observed to be sexually active (i.e. attempting to mate or copulate). Three of these nine sexually inactive males and nine of the 20 sexually inactive females achieved genetic parentage, which means that they copulated unnoticed and represent the subset of individuals that we did not observe.

### Female choice hypothesis

**Figure 3:**
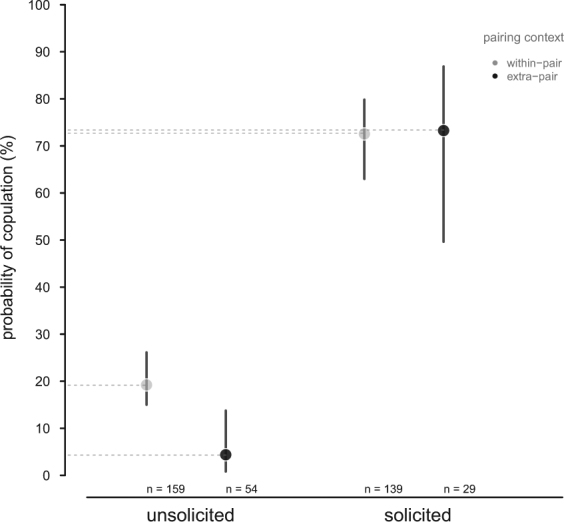
Mating attempts leading to copulation in house sparrows in relation to female solicitation and pairing status (*N* = 381 mating attempts). Female solicitation statistically significantly increased the likelihood of copulation. The effect depended on the pairing context: without female solicitation, copulations were more common with the social male than with an extra-pair male. Unsolicited (i.e. male-initiated) mating attempts were least successful. Filled dots represent posterior model means and the horizontal dashed lines were added to help visualisation. Vertical lines represent CrI.

We refer to a male initiated copulation as an unsolicited copulation in this manuscript. A within-pair mating attempt was about four-fold more likely and an extra-pair mating attempt about 17-fold more likely to lead to copulation when they were female-solicited compared to mating attempts that were unsolicited (Table 2, Fig. 3). Further, solicited within-pair and extra-pair copulations were equally common but only 4.3% of unsolicited extra-pair mating attempts led to copulation, compared with 19.1% of within-pair mating attempts (Table 2, Fig. 3). The ages of males were not associated with the success of extra-pair or within-pair mating attempts (Table 2). Additionally, the number of unsolicited extra-pair mating attempts was almost double that of solicited extra-pair mating attempts (54 male attempts *vs* 29 female attempts, binomial test *P* < 0.01, Fig. 3), while the numbers for within-pair mating attempts were more balanced between the sexes (159 male attempts *vs* 139 female attempts, binomial test *P* = 0.27, Fig. 3).

**Table 2:** Female solicitation had a statistically significant positive effect on whether a copulation occurred (*N* = 381 mating attempts).

	estimate (lower CrI to upper CrI)
**Fixed effects**	
(intercept)	−1.32 (−1.98 to −0.62)
solicited	**2.45** (**1.88 to 3)**
extra-pair	**−1.87** (**−3.45 to −0.28)**
male age	−0.03 (−0.36 to 0.30)
male age^2^	−0.05 (−0.41 to 0.29)
solicited * extra-pair	**1.84** (**0.01 to 3.65)**
aviary B	−0.27 (−1.04 to 0.54)
aviary C	0.16 (−0.98 to 0.57)
aviary D	−0.19 (−0.98 to 0.56)
**Random effects**	
male ID	0.17 (0.12 to 0.23)
female ID	0 (0 to 0)

In the absence of female solicitation, extra-pair copulations were statistically significantly less common than within-pair copulations. Results are from a GLMM with a binomial error distribution (logit-link function). Female solicitation (“solicited”, “unsolicited”) and pairing status (“within”- or “extra-pair”) were categorical fixed effects as well as the interaction of female solicitation and pairing status. Male age was centred and scaled and the outcome variable was a binary response of a mating attempt leading to copulation (“yes”, “no”). We show the model’s posterior means and CrI. CrIs interpreted as statistically significant are in bold.

### Extra-pair offspring as a proxy for extra-pair copulations

**Figure 4:**
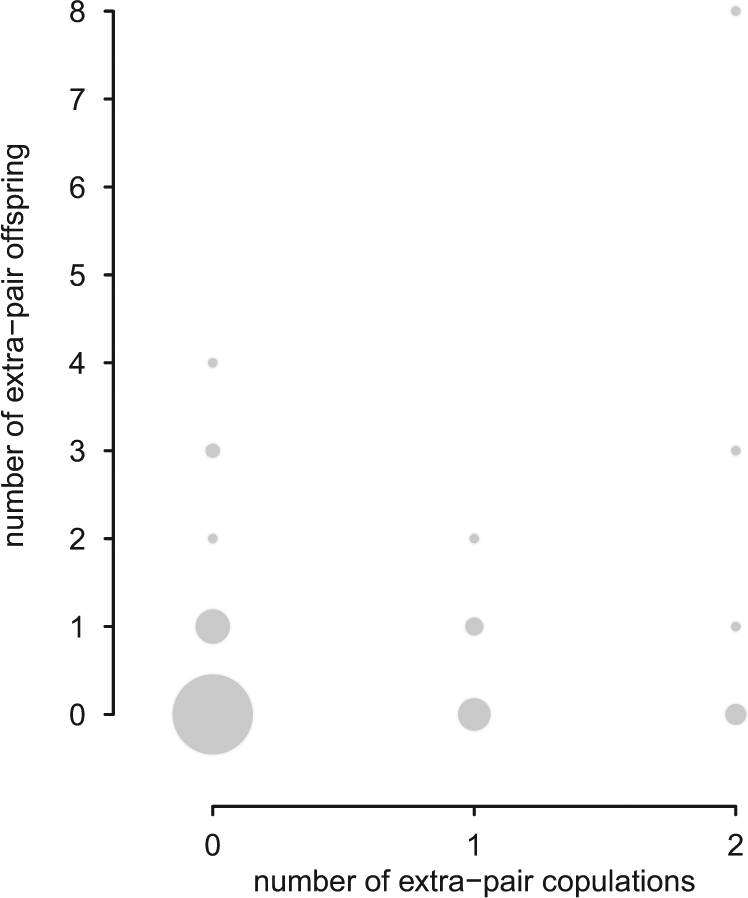
Individual data of house sparrow extra-pair copulations and extra-pair offspring (*N* = 85 males). The number of extra-pair copulations was not correlated with the number of extra-pair offspring.

The number of extra-pair copulations was not correlated with the number of extra-pair offspring (*N* = 85 males, Spearman rank correlation, *rho* = 0.15, *P* = 0.16, Fig. 4). Of the 85 males in this analysis, 55 males attempted extra-pair mating and 20 subsequently copulated with an extra-pair female compared to 53 out of 85 males that achieved within-pair copulations (Supplementary Information Fig. S1 for the correlation of within-pair copulations with within-pair offspring, Spearman rank correlation, *rho* = 0.33, *P* < 0.01). It does not seem reasonable to assume that extra-pair correlations correlate as strongly with paternity as within-pair copulations^25^. Still, we cannot exclude the possibility that the lack of correlation is driven by missing observations, so the results should be regarded as preliminary. There was no difference between the average age of males that were observed performing an extra-pair copulation (mean age 4.5 years, *N* = 20 males) and those that were not (mean age 4.6 years, *N* = 65 males, unpaired *t*-test *t*_36.54_ = 0.17, *P* = 0.87).

## Discussion

We find that middle-aged males, old birds in the wild^23^, produced most extra-pair offspring, which mirrors the results in a wild house sparrow population where extra-pair paternity increased with age in males before showing a decline^12^. In the wild, house sparrows that reach adulthood live on average for 3.4 years, and up to a maximum of 13 years^23^. The precise age of individuals is known in both studies, which allows extremely old males to be identified and a quadratic age effect on extra-pair paternity to be detected. Further, we did not find an association between extra- over within-pair mating and male age or female choice and male age. Our results imply that male age may not be an important predictor of extra-pair mating behaviour, and our results thus do not support the male manipulation hypothesis nor the female choice hypothesis.

Male age is the best predictor of extra-pair paternity in wild birds^1^, and our work confirms this in captivity, too. Male age and extra-pair mating behaviour were however, not associated and thus other mechanisms than mating behaviour could drive the relationship between extra-pair paternity and male age. Older males may outcompete younger males via post-copulatory mechanisms, for instance, if older males were better sperm competitors because of larger testes^26^. Alternatively, across iteroparous taxa, individuals show a peak in offspring production before reproductive senescence commences, due to better access to resources^27^ or simply because older individuals have more opportunities to encounter females^28^. As our study used a one-point-in-time sampling approach for all individuals, ensured an equal opportunity for males to encounter females and *ad libitum* access to crucial resources such as nest sites, nesting material and food, the statistically significant non-linear relationship between extra-pair paternity and male age could be the result of post-copulatory mechanisms that favoured fertilisation by older males.

Our study tested for a correlation of extra-pair mating with male age using, to our best knowledge for the first time, a communal breeding set-up of birds of known ages. In four populations, older males did not attempt nor achieve more extra-pair copulations than younger males. A possible limitation is that a competitive component to an old male mating advantage would have been reduced per individual with our set-up because we increased the number of old males (i.e. our populations did not represent the typical age pyramid found in wild populations: many young and few old individuals). Yet, we predicted an overall effect of male age on extra-pair mating behaviour and reducing the number of males at old ages experimentally would have reduced the chance of detecting a population effect.

Spatial proximity between territories, i.e. nest boxes as in our breeding design, eliminates costs of forays into neighbouring territories^13^ and creates opportunities for intrusion that could have elevated the frequency of extra-pair copulations for all males. With close proximity between territories, male pre-copulatory display will also reach multiple females simultaneously, which might further increase the frequency of extra-pair mating behaviour, and the proportion of extra-pair young^29^, but see^30^. However, even if extra-pair mating behaviour had been elevated by our captive set-up, we may have still underestimated the number of extra- (Fig. 4) and within-pair copulations (Supplementary Information Fig. S3). For instance, 14 males, out of a total of 65 males that were not seen performing extra-pair copulations, achieved extra-pair paternity. Hence, our observed copulation rates are lower than the true rate of copulations and the data should be regarded as preliminary. Second, if the unobserved males represent a non-random subsample, results might be biased. Captive studies have the advantage that males missing from observations are known and the number of males that we never observed as sexually active was small (10%). Importantly, the mean age of these males was similar to the mean age of males that we observed as sexually active, highlighting that our mating behaviour analyses in relation to male age were likely unbiased.

It is difficult to prove that females are making an active choice when choosing a mate^31^. In captivity, choice chamber tests are often used but these do not necessarily reflect female copulation behaviour (see^32^ for a summary). In the wild, extra-pair offspring are used as a proxy, but a bias towards older fathers does not necessarily mean that females prefer to copulate with older males. We combined the best of both approaches by allowing females to choose among multiple males of different ages and studying copulation behaviour directly. We found that female solicitation was not associated with male age (Supplementary Information Table S4). This contrasts with an experimental study where the social mates of western bluebird, *Sialia mexicana*, females were removed: subsequently, females were more likely to accept copulations from intruding males older than their own, absent mate^22^. Differences are anticipated even within species because females will vary in their impetus to copulate promiscuously^33^. While our study does not reveal which traits, if any, females prefer in males, it suggests that male age does not predict whether females solicit copulations or not.

Mating attempts were statistically significantly more likely to succeed when females solicited males. In species where males lack intromittent organs it is expected that female cooperation is important for copulations^34^. Also, greater female cooperation towards within-pair than to extra-pair mating has been shown before, e.g.^35^ but see^18,36^. Our study takes these findings a step further by showing that the likeliness of a copulation is most dependent on whether it was solicited by a female, not just her cooperation, especially so for extra-pair copulations. We also observed that males, not females, mostly initiated extra-pair mating attempts, which makes sense as the incentive for a female to cheat is lower than for a male, yet only 4.3% of these unsolicited extra-pair mating attempts led to copulation. Despite their markedly reduced success, the probability of an unsolicited extra-pair mating succeeding in copulation was between 0.8–14% (Fig. 3), which would be enough for selection to act upon, given that the behaviour is linked to reproductive success. In several species, extra-pair copulations have been found to not result in extra-pair paternity^37–39^ but this was not the case in our study. It would be informative to compare the fertilisation efficiency of solicited versus unsolicited extra-pair copulations in future work.

Females actively solicited promiscuous copulations but, in contrast, convenience polyandry, i.e. females giving in to extra-pair males^13^, seems to play a minor role in house sparrows. Female extra-pair behaviour could be explained by indirect selection for alleles that increase male promiscuity^40^. Intriguingly, while copulations initiated by females were more successful than those initiated by males, the former were not always successful: approximately 25% of solicitations did not result in a cloacal kiss. There were multiple reasons for mating attempts failing, such as the clumsiness of the couple, or disturbance by conspecifics, which corroborates observations on mating behaviour in zebra finches^40^. We also witnessed males ignoring female solicitation. A male’s refusal to copulate might be explained by a physiological constraint in house sparrows because males can become ejaculate-depleted within a day^41^. It would be interesting to quantify and better understand the occurrence of male resistance to female mating attempts in future studies.

Our study showed that extra-pair paternity is unlikely to predict extra-pair copulations well, given that male initiated extra-pair mating attempts were mostly unsuccessful. Also, one could expect the relationship between extra-pair copulations and extra-pair paternity to be weaker compared to that between within-pair copulations and within-pair paternity^25^. A lack of a relationship between extra-pair copulations and paternity is, however, biologically implausible and future work should reveal the strength of relationship between extra-pair mating and extra-pair paternity.

To conclude, the observation that females responded more cooperatively to copulation attempts by their social male than by an extra-pair male also emphasises a function of the pair-bond that precedes biparental care. Females also solicited extra-pair copulations, highlighting that extra-pair courtship, despite being male-driven, is a mating strategy adopted by both sexes, the success of which is mainly under female control in house sparrows. Extra-pair copulation will allow some males to increase their reproductive success, and post-copulatory mechanisms might be responsible for the robust correlation between extra-pair paternity and male age in birds.

## Methods

### Study population and experimental breeding set-up

Birds were kept at the Max Planck Institute for Ornithology in Seewiesen, Germany and looked after as described in^42^. The population consisted of wild-caught house sparrows born in 2005 and 2006 and their offspring born in captivity. Males and females were assigned to four aviaries each measuring 3.6 m × 4.0 m × 2.2 m avoiding individuals that were assigned to the same breeding aviary in the previous year and relatives. Per aviary, we had a similar number of males and females, between 21 and 24 pairs, at equal sex ratios and uniform age distributions, and there was no evidence for age-assortative mating in our four populations (*N* = 75 social pairs, Spearman rank correlation, *rho* = −0.05, *P* = 0.66). Birds were between one and ten years old but we lacked males aged two, four and six to seven years (see Supplementary Information Table S5 for age structures and densities per aviary).

House sparrows are hole-nesting passerines that use nest boxes for breeding^43^, so all aviaries were equipped with sufficient individually marked nest boxes to accommodate the respective numbers of pairs plus one extra nest box to reduce competition for sites, e.g. 22 nest boxes for an aviary that held 21 pairs of birds. All birds had *ad libitum* access to food and water^42^, and to nesting material such as hay, horse hair and coconut fibre. Further, each bird was equipped with a combination of a uniquely-numbered metal ring and three coloured plastic rings to allow identification.

### Paternity analysis

Nest boxes were monitored daily. Five to seven days after females initiated incubation, we collected all eggs for parentage analysis, and replaced eggs with fake plaster eggs, resembling house sparrow eggs, to retain natural breeding sequences. We used 12 microsatellite markers^44^ (*Ase18*, *Pdo1*, *Pdo3, Pdo5, Pdo6, Pdo9, Pdo10*, *Pdo16*, *Pdo17, Pdo19*, *Pdo22*, *Pdo27*) and the procedures described in^44^ for genotyping. Cervus version 3.0.7^45^ was used to establish genetic parentage. We first assigned putative mothers from behavioural observations and then, in a second step, we used the confirmed maternity and allowed for all males per aviary to be sires to determine paternity. Of 405 embryos, 400 could be assigned to genetic sires with 95% confidence. For the remaining five embryos, genetic paternity could not be established.

### Behavioural observations

Behavioural observations of 174 individuals were made daily from 15 April – 18 June 2015, which represents the beginning and the middle of house sparrow’s breeding season^23^ and allowed females to have a minimum of two broods, with seven females out of 87 completing a third brood. Daily behavioural observations were started between 07:00–07:30 and were recorded live using a Zeiss Victory 10 × 42 mm binocular mostly by observer CWCT. Three co-workers substituted the main observer (CWCT) on six successive days. Our decision to always start behavioural observations at dawn was informed by the observation that house sparrow displays and copulations and extra-pair displays peak during that time^46,47^. Observers were blind with regard to the age and pairing status of individuals when recording birds’ behaviour. As the four aviaries were too large to be observed with an unobstructed view, we divided each aviary into three same-sized sections (Supplementary Information Figs S2 and S3). Each aviary section was observed separately for 10 to 15 minutes resulting in a total observation time of two to three hours per day. The order of the observations of each aviary section was randomised, using the function “sample” in R version 3.2.1^48^, to ensure that observations were not compromised by potential order effects. We identified pair-bonds and nest box owners by which birds were seen repeatedly at or in each nest box, attending and building nests, and which birds laid and incubated eggs. These criteria were sensible because house sparrows do not engage in pair-bond formation behaviour such as allopreening^23^. Instead, house sparrows commonly initiate pair-bonds after a male has procured a nest site, and the repeated presence of a male and a female at the nest is a strong indication of their pair-bond^23^.

We also observed individual copulation behaviour. A male house sparrow displays by approaching a female, lifting his wings slightly, hopping around her vigorously, and vocalising continuously before attempting to mount her^49^. Male house sparrows can also attempt copulation during communal chases of a single female but these chases, while vigorous, rarely result in successful copulations^23^. When females initiate copulation, they adopt a crouching position with their wings quivering and their posterior end held upright (see the video file in the Supplementary Information). This female behaviour is referred to as solicitation and is distinct from a female’s passive cooperation in a male initiated copulation (i.e. raising her tail and leaning forward to accept a male mating attempt)^49^. We refer to a male initiated copulation as an unsolicited copulation in this manuscript. We recorded both a male display and a female solicit, together with the identities of the individuals involved. Subsequently, we recorded whether solicited or unsolicited mating attempts were successful, i.e. resulted in copulation, where a male mounted a female and both individuals bent their tails for a cloacal kiss^50^. In house sparrows, mating behaviour involves copulation bouts comprised of repeated rapid mountings that do or do not include cloacal contact^23^. The adaptive significance of copulation bouts is not well understood but their occurrence outside the breeding season highlights that, apart from fertilisation, repeated mounting might be important for pair formation^23^. We used the number of copulation bouts comprising at least one copulation rather than the number of mountings, together with the identities of individuals, in subsequent analyses of whether mating took place within or outside of a pair.

### Ethical note

This study was approved by the Government of Bavaria (Nr 311.5-5682.1/1-2014-024) and the Animal Care and Ethics Committee of the Max Planck Institute for Ornithology. Further, behavioural observations and animal husbandry were carried out in accordance to the directives 2010/63/EU of the European Parliament on the protection of animals used for scientific purposes.

### Statistical analyses

We used generalised linear models (GLM) and generalised linear mixed effects models (GLMMs,) with a binomial error distribution and a logit-link function to test the questions outlined below. In all models, male age was added as continuous mean centred and scaled explanatory variable, so that the variable male age was measured in the unit of standard deviations (sd) from the mean. Aviary identity was included as a fixed effect in all analyses because with only four levels it could not be fitted as a random effect^51^.

- *Male age and its association with extra-pair paternity*
  Using a GLM with binomial errors, we tested whether male age (explanatory variable) positively predicted extra-pair paternity by fitting the number of extra-pair offspring as a proportion (extra-pair out of total offspring) response variable. We used a proportional response variable rather than a Poisson GLM because we were not interested whether a single offspring was extra-pair or within-pair, but in the frequency of males to sire extra-pair offspring. Also, the number of extra-pair offspring was low (overall mean 0.10 extra-pair offspring/offspring) and to adjust for the effect that males that achieve higher paternity inevitably have higher detection rates of extra-pair paternity^52^. As the relationship between extra-pair paternity and male age was expected to be non-linear^12^, we added a quadratic age term as an explanatory variable to the model. We excluded 11 males that were unpaired and thus could be considered floaters^53^. However, qualitatively, the results remained similar to when floaters were included (Supplementary Information Table S6). The total sample size, excluding floaters, was 75 males.
- *Male manipulation hypothesis*
  Here, we assessed whether male extra-pair mating behaviour was positively associated with male age (explanatory variables fitted as a linear and quadratic age term) by using two proportional response variables in a binomial GLM. The first response variable was the proportion of a male’s extra mating attempts. We excluded two outliers that caused overdispersion^54^ but first established that this decision did not mediate our analysis by re-running the analysis including the two outliers and confirming that the results were qualitatively similar. The second response variable was the proportion of a male’s extra-pair copulations. For both analyses, we again excluded 11 male floaters^53^ but the results remained similar when floaters were included (Supplementary Information Table S7). Also, four males were paired to two females simultaneously, they were polygynous. For the latter males, we summed the mating attempts and copulations for both their pair-bonds and only considered mating attempts and copulations outside their two pair-bonds as extra-pair. Note, that including a quadratic age term showed no effect and was therefore not included in the final models. The total sample size, without floaters, was 75 males for the mating attempts GLM and 74 males for the copulation GLM.
- *Female choice hypothesis*
  To assess how female choice affects the likelihood of copulation, we fitted the probability of whether a mating attempt led to copulation (“yes” or “no”) as a response variable in a GLMM with a binary response. Female solicitation (“solicited”, “unsolicited”) and pairing context (“within”- or “extra-pair”) were categorical explanatory variables as well as the interaction between both. Male age was added as an explanatory variable, including a quadratic age term. Having both female solicitation and male age as predictors in the same model was justified because there was no association between male age and female solicitation behaviour (Supplementary Information Table S4), which implies that the effects can be interpreted independently from each other and the analyses did not suffer from collinearity. We excluded five floaters present in this dataset, but the analysis including male floaters yielded similar results (Supplementary Information Table S8). Again, only mating attempts and copulations outside both pair-bonds for polygynous males were considered to be extra-pair. The total sample size was 381 copulations attempts involving 71 males, excluding floaters. As repeated measures were obtained across males and females, male and female IDs were added as a random intercept.
- *Extra-pair offspring as a proxy for extra-pair copulations*

Finally, we tested whether the number of observed extra-pair offspring was correlated with the number of observed extra-pair copulations using a Spearman rank correlation test.

We used R version 3.4.1^48^ and the package “lme4”^55^ to run models. We then used the package “arm” and the function “sim” to simulate values from the posterior distributions (*N* = 2000 draws) of the model parameters from lme4, assuming non-informative priors. From the simulated values, we extracted 95% Credible Intervals (CrI), based on the 2.5% and 97.5% quantiles of the posterior distributions^56^. The CrI represent the uncertainty of our estimates but we also used them for statistical significance testing because CrI not overlapping zero can be interpreted as a Frequentist p-value < 0.05^54^. For all models, we followed the recommendation in^54,57^ to ensure that model assumptions and fit were met, including checking for overdispersion and multi-collinearity.

### Data availability

All datasets are available at the Open Science Framework (10.17605/OSF.IO/FYURG).

## Electronic supplementary material

Supplementary Info

## References

[1] Cleasby IR, Nakagawa S (2012). The influence of male age on within-pair and extra-pair paternity in passerines. Ibis (Lond. 1859)..

[2] Hsu YH, Schroeder J, Winney I, Burke T, Nakagawa S (2015). Are extra-pair males different from cuckolded males? A case study and a meta-analytic examination. Mol. Ecol..

[3] Akcay E, Roughgarden J (2007). Extra-pair paternity in birds: review of the genetic benefits. Evol. Ecol. Res..

[4] Griffith SC, Owens IP, Thuman KA (2002). Extra pair paternity in birds: a review of interspecific variation and adaptive function. Mol. Ecol..

[5] Hansen TF, Price DK (1995). Good genes and old age: Do old mates provide superior genes?. J. Evol. Biol..

[6] Forstmeier W, Nakagawa S, Griffith SC, Kempenaers B (2014). Female extra-pair mating: adaptation or genetic constraint?. Trends Ecol. Evol..

[7] Weatherhead PJ, Boag PT (1995). Pair and extra-pair mating success relative to male quality in red-winged blackbirds. Behav. Ecol. Sociobiol..

[8] Wetton JH, Burke T, Parkin DT, Cairns E (1995). Single-Locus DNA-Fingerprinting reveals that male reproductive success Increases with age through extra-pair paternity in the house sparrow (*Passer domesticus*). Proc. R. Soc. B Biol. Sci..

[9] Sundberg J, Dixon A (1996). Old, colourful male yellowhammers, *Emberiza citrinella*, benefit from extra-pair copulations. Anim. Behav..

[10] Tarof SA, Kramer PM, Tautin J, Stutchbury BJM (2012). Effects of known age on male paternity in a migratory songbird. Behav. Ecol..

[11] Gonzalez-Solis J, Becker PH (2002). Mounting frequency and number of cloacal contacts increase with age in common terns *Sterna hirundo*. J. Avian Biol..

[12] Hsu YH (2017). Age-dependent trajectories differ between within-pair and extra-pair paternity success. J. Evol. Biol..

[13] Westneat DF, Stewart IRK (2003). Extra-pair paternity in birds: causes, correlates, and conflict. Annu. Rev. Ecol. Evol. Syst..

[14] Dixon A, Ross D, Omalley SLC, Burke T (1994). Paternal investment is inversely related to degree of extra-pair paternity in the reed bunting. Nature.

[15] Morales MB, Alonso JC, Martín C, Martín E, Alonso J (2003). Male sexual display and attractiveness in the great bustard*Otis tarda*: the role of body condition. J. Ethol..

[16] Possamai CB, Young RJ, De Oliveira RCR, Mendes SL, Strier KB (2005). Age-related variation in copulations of male northern muriquis (*Brachyteles hypoxanthus*). Folia Primatol..

[17] Shively C, Clarke S, King N, Schapiro S, Mitchell G (1982). Patterns of sexual behavior in male macaques. Am. J. Primatol..

[18] Wagner RH (1992). Extra-pair copulations in a lek: the secondary mating system of monogamous razorbills. Behav. Ecol. Sociobiol..

[19] Kokko H, Lindstrom J (1996). Evolution of female preference for old mates. Proc. R. Soc. B Biol. Sci..

[20] Møller AP, Ninni P (1998). Sperm competition and sexual selection: a meta analysis of paternity studies of birds. Behav. Ecol. Sociobiol..

[21] Brooks R, Kemp DJ (2001). Can older males deliver the good genes?. Trends Ecol. Evol..

[22] Dickinson JL (2001). Extrapair copulations in western bluebirds (*Sialia mexicana*): Female receptivity favors older males. Behav. Ecol. Sociobiol..

[23] Anderson, T. R. *Biology of the ubiquitous house sparrow. From genes to populations, chapter 4*. (Oxford University Press, 2006).

[24] Hsu YH, Schroeder J, Winney I, Burke T, Nakagawa S (2014). Costly infidelity: low lifetime fitness of extra-pair offspring in a passerine bird. Evolution (N. Y)..

[25] Dunn PO, Lifjeld JT (1994). Can extra-pair copulations be used to predict extra-pair paternity in birds?. Anim. Behav..

[26] Laskemoen T, Fossøy F, Rudolfsen G, Lifjeld JT (2008). Age-related variation in primary sexual characters in a passerine with male age-related fertilization success, the bluethroat *Luscinia svecica*. J. Avian Biol..

[27] Lack D (1968). Ecological adaptations for breeding in birds. Science.

[28] Jensen H (2004). Lifetime reproductive success in relation to morphology in the house sparrow *Passer domesticus*. J. Anim. Ecol..

[29] Richardson DS, Burke T (2001). Extrapair paternity and variance in reproductive success related to breeding density in Bullock’s orioles. Anim. Behav..

[30] Westneat DF, Sherman PW (1997). Density and extra-pair fertilizations in birds: A comparative analysis. Behav. Ecol. Sociobiol..

[31] Dunbar, R. I. M. Life history tactics and alternative strategies of reproduction. In *Mate choice* (ed. Bateson, P.) 423–447 (Cambridge University Press, 1983).

[32] Ihle, M., Kempenaers, B. & Forstmeier, W. Fitness benefits of mate choice for compatibility in a socially monogamous species. *PLoS Biol*. **13** (2015). 10.1371/journal.pbio.1002248 PMC4569426 26366558

[33] Forstmeier, W. Do individual females differ intrinsically in their propensity to engage in extra-pair copulations? *PLoS One* **2** (2007). 10.1371/journal.pone.0000952 PMC1978515 17895992

[34] Briskie JV, Montgomerie R (1997). Sexual selection and the intromittent organ of birds. J. Avian Biol..

[35] Westneat DF (1987). Extra-pair copulations in a predominantly monogamous bird: observations of behaviour. Anim. Behav..

[36] Frederick PC (1986). Extrapair copulations in the mating system of white ibis (*Eudocimus albus*). Behavior.

[37] Sanchez-Macouzet O, Rodriguez C, Drummond H (2014). Better stay together: pair bond duration increases individual fitness independent of age-related variation. Proc. R. Soc. B Biol. Sci..

[38] Hunter FM, Burke T, Watts SE (1992). Frequent copulation as a method of paternity assurance in the northern fulmar. Anim. Behav..

[39] Schwartz MK (1999). Female-solicited extrapair matings in Humboldt penguins fail to produce extrapair fertilizations. Behav. Ecol..

[40] Forstmeier W, Martin K, Bolund E, Schielzeth H, Kempenaers B (2011). Female extrapair mating behavior can evolve via indirect selection on males. Proc. Natl. Acad. Sci..

[41] Birkhead TR, Veiga JP, Møller AP (1994). Male sperm reserves and copulation behavior in the house sparrow, *Passer domesticus*. Proc. R. Soc. B Biol. Sci..

[42] Girndt A (2017). Method matters: Experimental evidence for shorter avian sperm in faecal compared to abdominal massage samples. PLoS One.

[43] Sánchez-Tójar, A. *et al*. Winter territory prospecting is associated with life-history stage but not activity in a passerine. *J. Avian Biol*. **48** (2016).

[44] Dawson DA (2012). Microsatellite resources for Passeridae species: a predicted microsatellite map of the house sparrow *Passer domesticus*. Mol. Ecol. Resour..

[45] Kalinowski ST, Taper ML, Marshall TC (2007). Revising how the computer program CERVUS accommodates genotyping error increases success in paternity assignment. Mol. Ecol..

[46] Møller AP (1987). House sparrow, *Passer domesticus*, communal displays. Anim. Behav..

[47] Schlicht L, Kempenaers B (2016). Courtship calls in Blue Tits *Cyanistes caeruleus*: daily and seasonal occurrence and link to paternity. Ardea.

[48] R Development Core Team (2013). R: A language and environment for statistical computing.

[49] Summers-Smith D (1955). Display of the house sparrow *Passer domesticus*. Ibis (Lond. 1859)..

[50] Nyland KB, Lombardo MP, Thorpe PA (2003). Left-sided directional bias of cloacal contacts during house sparrow copulations. Wilson Bull..

[51] Bolker BM (2009). Generalized linear mixed models: a practical guide for ecology and evolution. Trends Ecol. Evol..

[52] Burley, N. T. & Parker, P. G. Emerging themes and questions in the study of avian reproductive tactics. In *Avian reproductive tactics: Female and male perspectives* (eds Burley, N. T. & Parker, P. G.) 1–20 (Amerian Ornithologist’s Union, 1998).

[53] Smith JNM, Arcese P (1989). How fit are floaters? Consequences of alternative territorial behaviors in a nonmigratory sparrow. Am. Nat..

[54] Korner-Nievergelt, F. *et al*. *Bayesian data analysis in ecology using linear models with R, BUGS, and Stan*. (Academic Press, 2015).

[55] Bates D, Machler M, Bolker BM, Walker SC (2015). Fitting Linear Mixed-Effects Models using lme4. J. Stat. Softw..

[56] Gelman, A. & Hill, J. *Data analysis using regression and multilevel/hierarchical models*. (Cambridge University Press, 2007).

[57] Zuur, A. F., Ieno, E. N., Walker, N. J., Saveliev, A. A. & Smith, G. M. *Mixed effects models and extensions in ecology with R*. **1**, (Springer-Verlag, 2009).

