# Supplementary material for: Male age is associated with extra-pair paternity, but not with extra-pair mating behaviour
Source: Sci Rep. 2018 May 30;8:8378. doi: 10.1038/s41598-018-26649-1 (PMC5976671; doi:10.1038/s41598-018-26649-1)
Supplement: Supplementary file 1 — Supplementary Info [file 41598_2018_26649_MOESM1_ESM.pdf]

Male age is associated with extra-pair paternity, but not with extra-pair mating behaviour

Antje Girndt[a,b,c*], Charlotte Wen Ting Chng[b], Terry Burke[d], Julia Schroeder[a,b]

[a]Evolutionary Biology, Max Planck Institute for Ornithology, Seewiesen, Germany

[b]Department of Life Sciences, Imperial College London, Silwood Park Campus, Ascot, United Kingdom

[c]International Max-Planck Research School (IMPRS) for Organismal Biology, University of Konstanz, Konstanz, Germany

[d]Department of Animal and Plant Sciences, University of Sheffield, Sheffield, United Kingdom

*** Correspondence:**

Antje Girndt

a.girndt@gmail.com

SUPPLEMENTARY INFORMATION

Table S1.

	estimate (standard error)	*z - value*	*P - value*
**Fixed effects**			
(intercept)	1.04 (0.32)	3.25	0.001
male age	0.92 (0.37)	2.51	**0.01**
male age^2	-0.08 (0.29)	-0.27	0.79
aviary B	-0.39 (0.51)	-0.76	0.45
aviary C	-1.75 (0.59)	-3	**0.003**
aviary D	-2.04 (0.85)	-2.41	**0.02**

Extra-pair paternity showed a statistically significant linear relationship with male age (N = 75 males). Results are from a zero-inflated Poisson (log-link function) using the package "pscl" [1]. Male age was centred and scaled. Statistically significant results are in bold.

Table S2.

a)

	estimate (lower CrI to upper CrI)
**Fixed effects**	
(intercept)	-1.24 (-1.76 to -0.69)
male age	0.07 (-0.19 to 0.33)
male age^2	-0.07 (-0.40 to 0.26)
aviary B	0.30 (-0.37 to 0.95)
aviary C	0.06 (-0.66 to 0.72)
aviary D	-0.13 (-0.82 to 0.54)
**b)**	
**Fixed effects**	
(intercept)	-1.08 (-1.94 to -0.27)
male age	0.03 (-0.51 to 0.57)
male age^2	-0.38 (-0.97 to 0.20)
aviary B	-0.34 (-1.58 to 0.93)
aviary C	0.22 (-0.80 to 1.28)
aviary D	-1.04 (-2.33 to 0.30)

Neither the proportion of extra-pair mating attempts (a) (N = 73 males) nor the proportion of extra-pair copulations (b) (N = 74 males) was explained by the age of male house sparrows, *Passer domesticus*, excluding floaters [2]. Results are from a generalised linear model, GLM, assuming a binomial error distribution (logit-link function). Male age was centred and scaled. A) Extra- to within-pair mating attempts and b) extra- to within-pair copulations were fitted as a proportional response variable. We show the model's posterior means and CrI. CrIs interpreted as statistically significant are in bold.

Table S3.

a)

	estimate (lower CrI to upper CrI)
**Fixed effects**	
(intercept)	1.76 (1.52 to 1.98)
male age	-0.04 (-0.15 to 0.08)
male age^2	-0.07 (-0.20 to 0.06)
aviary B	-0.12 (-0.39 to 0.18)
aviary C	0.11 (-0.16 to 0.38)
aviary D	0.17 (-0.10 to 0.43)
**b)**	
**Fixed effects**	
(intercept)	0.87 (0.50 to 1.21)
male age	-0.02 (-0.19 to 0.17)
male age^2	-0.12 (-0.34 to 0.10)
aviary B	**-0.63 (-1.17 to -0.09)**
aviary C	0.13 (-0.31 to 0.56)
aviary D	0.25 (-0.18 to 0.66)

Neither the total number of mating attempts (a) (N = 73 males) nor the total number of copulations (b) (N = 74 males) was explained by the age of male house sparrows, excluding floaters. Results are from a GLM, assuming a Poisson error distribution (log-link function). Male age was centred and scaled. We show the model's posterior means and CrI. CrIs interpreted as statistically significant are in bold.

Table S4.

	estimate (lower CrI to upper CrI)
**Fixed effects**	
(intercept)	-0.16 (-0.75 to 0.44)
male age	0.13 (-0.16 to 0.43)
male age^2	-0.13 (-0.44 to 0.18)
aviary B	-0.56 (-1.32 to 0.12)
aviary C	0.09 (-0.64 to 0.84)
aviary D	0.31 (-0.39 to 1.07)
**Random effects**	
male ID	0.42 (0.30 to 0.54)

Male age (N = 77 males) did not explain the probability of solicitation (449 observations) in house sparrows, excluding floaters. Results are from a generalised linear mixed effect model, GLMM, assuming a binomial error distribution (logit-link function). Male age was centred and scaled and the outcome variable was a binary response of solicitation (“yes”, “no”). We show the model’s posterior means and CrI. CrIs interpreted as statistically significant are in bold.

Table S5.

	**aviary A**		**aviary B**		**aviary C**		**aviary D**	
	**males**	**females**	**males**	**females**	**males**	**females**	**males**	**females**
age in years	$N =$	$N =$	$N =$	$N =$	$N =$	$N =$	$N =$	$N =$
1	8	8	8	9	8	5	7	6
2	0	0	0	0	0	2	0	0
3	2	2	2	5	2	4	3	5
5	6	6	6	4	4	4	5	5
8-10	5	5	8	6	7	6	6	5
total number	21	21	24	24	21	21	21	21

We aimed at similar sample sizes per age and sex in our four house sparrow populations: young breeders (one to three years old), middle-aged breeders (five-year-old) and old breeders (eight to ten years old).

Table S6.

	estimate (lower CrI to upper CrI)
**Fixed effects**	
(intercept)	-1.29 (-1.88 to -0.70)
male age	**0.69 (0.24 to 1.15)**
male age^2	**-0.57 (-1.04 to -0.11)**
aviary B	-0.01 (-0.90 to 0.90)
aviary C	-0.58 (-1.61 to 0.43)
aviary D	**-1.33 (-2.36 to -0.30)**

The proportion of extra-pair paternity in relation to the age of male house sparrows, including floaters, showed a significant quadratic relationship with male age (N = 86 males). Results are from a generalised linear model, GLM, assuming a binomial error distribution (logit-link function). Male age was centred and scaled. Extra-pair to within-pair offspring was fitted as a proportional response variable. We show the model's posterior means and 95% Credible Intervals (CrI). CrIs interpreted as statistically significant are in bold.

Table S7.

a)

	estimate (lower CrI to upper CrI)
**Fixed effects**	
(intercept)	-1.21 (-1.74 to -0.68)
male age	0.16 (-0.09 to 0.41)
male age^2	0.01 (-0.31 to 0.31)
aviary B	0.23 (-0.44 to 0.91)
aviary C	0.05 (-0.59 to 0.73)
aviary D	-0.15 (-0.81 to 0.52)

b)

	estimate (lower CrI to upper CrI)
**Fixed effects**	
(intercept)	-1.08 (-1.93 to -0.24)
male age	0.03 (-0.50 to 0.59)
male age^2	-0.38 (-0.98 to 0.18)
aviary B	-0.34 (-1.63 to 0.99)
aviary C	0.22 (-0.90 to 1.28)
aviary D	-1.04 (-2.35 to 0.23)

Neither the proportion of extra-pair mating attempts (a) (N = 84 males) nor the proportion of extra-pair copulations (b) (N = 85 males) was explained by the age of male house sparrows, including floaters. Results are from a GLM, assuming a binomial error distribution (logit-link function). Male age was centred and scaled. A) Extra- to within-pair mating attempts and b) extra- to within-pair copulations were fitted as a proportional response variable. We show the model's posterior means and CrI. CrIs interpreted as statistically significant are in bold.

Table S8.

	estimate (lower CrI to upper CrI)
**Fixed effects**	
(intercept)	-1.32 (-2 to -0.64)
solicited	**2.44 (1.88 to 2.98)**
extra-pair	**-1.94 (-3.45 to -0.46)**
male age	-0.04 (-0.38 to 0.28)
male age^2	-0.04 (-0.40 to 0.29)
solicited * extra-pair	**1.77 (0 to 3.53)**
aviary B	-0.27 (-1.08 to 0.60)
aviary C	-0.02 (-0.83 to 0.82)
aviary D	-0.19 (-0.94 to 0.66)
**Random effects**	
male ID	0.22 (0.16 to 0.30)
female ID	0 (0 to 0)

Female solicitation had a significant positive effect on whether a copulation occurred in house sparrows, including floaters (N = 391 mating attempts). In the absence of female solicitation, extra-pair copulations were significantly less common than within-pair copulations. Results are from a GLMM with a binomial error distribution (logit-link function). Female solicitation ("solicited", "not solicited") and pairing status ("within"- or "extra-pair") were categorical fixed effects as well as the interaction of female solicitation and pairing status. Male age was centred and scaled and the outcome variable was a binary response of a mating attempt leading to copulation ("yes", "no"). We show the model's posterior means and CrI. CrIs interpreted as statistically significant are in bold.

Figure S1.

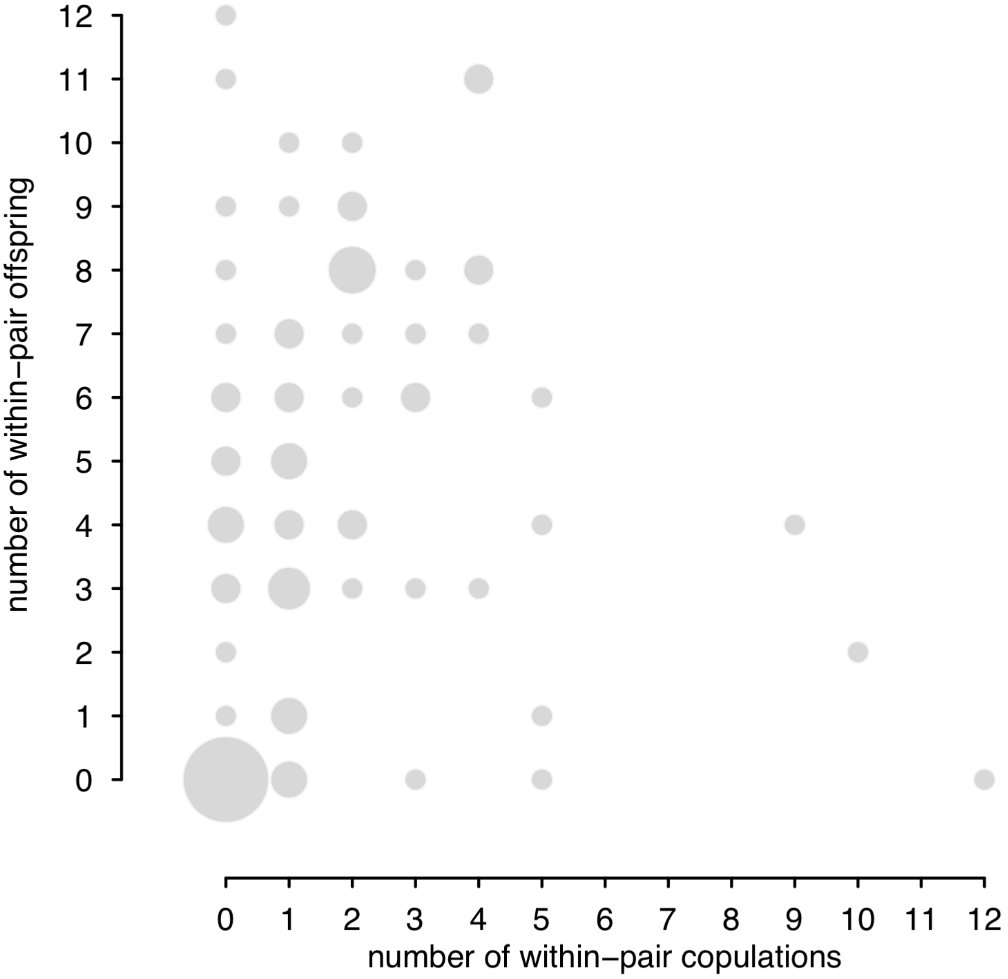

Individual data of within-pair copulations and within-pair offspring (*N* = 85 males). The number of within-pair copulations was correlated with the number of within-pair offspring (Spearman rank correlation, *rho* = 0.33, $P < 0.01$)

Figure S2. Schematic unscaled bird's eye view of two of the four house sparrow aviaries. Numbered squares illustrate nest boxes. Red-bordered squares are nest boxes that were fitted above each other, displaced by 30 cm. For example, in aviary B, nest box 99 was fitted 30 cm above nest box 97. Vertical bold lines represent aviary walls. Vertical interrupted lines highlight single open sections within each aviary. The dashed lines represent the outer-wall that was covered with mesh wire. Observations were performed daily through the window into each individual section from 15 April – 18 June 2015, which represents the beginning and the middle of house sparrow's breeding season [3].

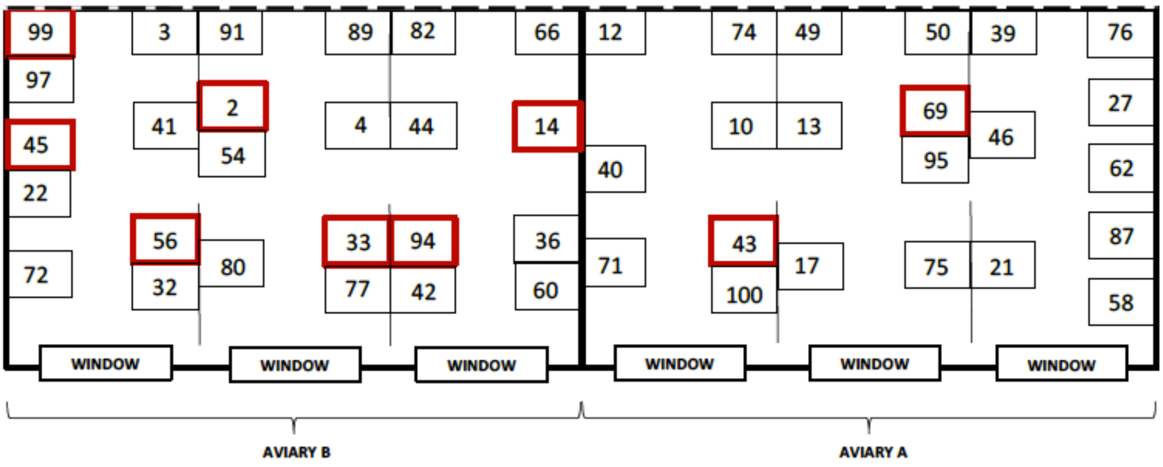

Figure S3. Example view of one house sparrow aviary section. Observations were performed in close proximity to the aviary section window but in contrast to the photograph the observer could see the whole aviary section and not just the upper part. Photograph courtesy of Charlotte Wen Ting Chng.

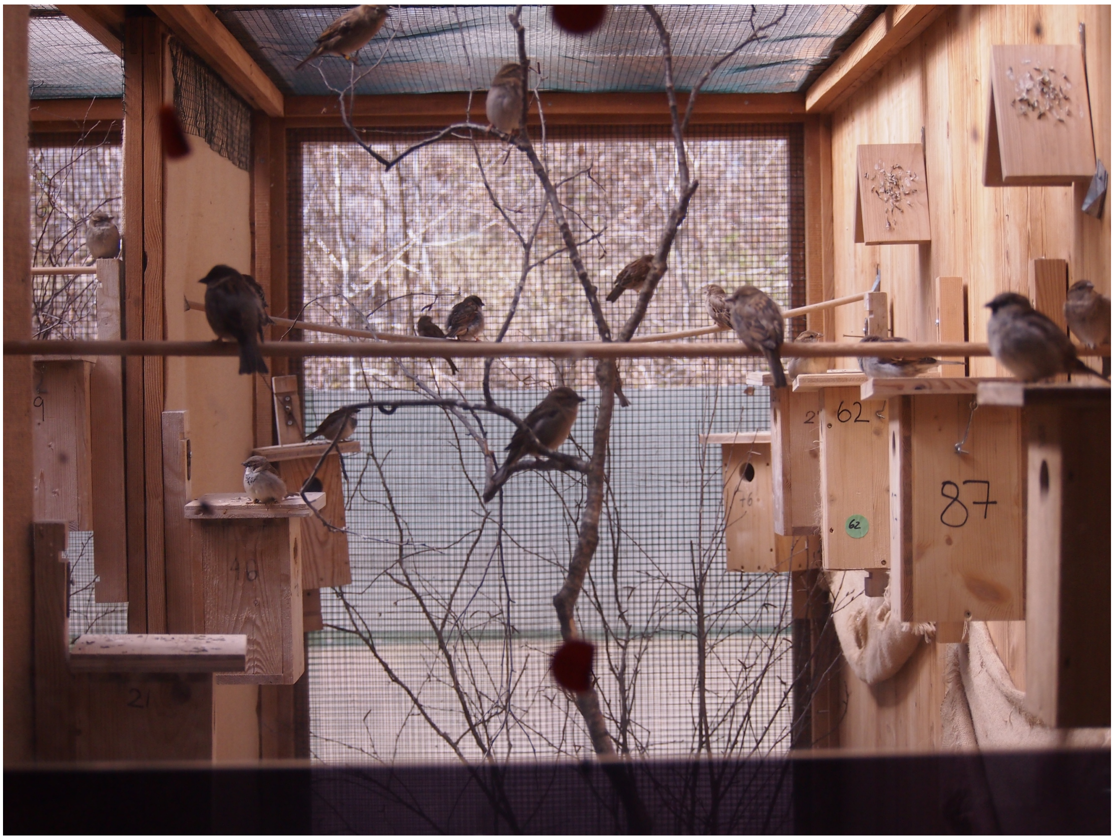

References

1. Jackman, S. pscl: Classes and Methods for R Developed in the Political Science Computational Laboratory, Stanford University. *http://www.pscl.stanford.edu/* R package version 1.04.1 (2011).
2. Smith, J. N. M. & Arcese, P. How fit are floaters? Consequences of alternative territorial behaviors in a nonmigratory sparrow. *Am. Nat.* **133,** 830–845 (1989).
3. Anderson, T. R. *Biology of the ubiquitous house sparrow. From genes to populations, chapter 4*. (Oxford University Press, 2006).
